# Drug-Target Interaction Network Analysis of Gene-Phenotype Connectivity Maintained by Genistein

**DOI:** 10.1089/cmb.2019.0443

**Published:** 2020-12-04

**Authors:** Baoshan Li, Yi Jiang, Jingxin Chu, Qian Zhou

**Affiliations:** ^1^ Department of General Medicine and Geriatrics, Chongqing University Central Hospital/Chongqing Emergency Medical Center, Chongqing, China.; ^2^ Key Laboratory of Molecular Biology for Infectious Diseases, Ministry of Education, Chongqing, China.; ^3^ Institute for Viral Hepatitis, Chongqing Medical University, Chongqing, China.; ^4^ Department of Infectious Diseases, The Second Affiliated Hospital, Chongqing Medical University, Chongqing, China.

**Keywords:** drug-target interaction network, gene-phenotype connectivity, genistein, HBV-related liver cancers

## Abstract

Genistein is a type of isoflavone, which has been widely described as an antitumor agent in many cancers. The present study aimed to provide information on the mechanisms of genistein's activity and thus enable a wider range of targeted therapies in hepatitis B virus (HBV)-related liver cancer. We searched the DrugBank database for direct targets of genistein, which were then analyzed through the STRING (Search Tool for the Retrieval of Interacting Genes/Proteins) database to predict their secondary protein targets. Thirteen primary protein targets of genistein and 209 secondary protein targets-associated genes were identified. The data were integrated into the network of protein targets-associated genes and visualized with the Cytoscape software. We further carried out GO (Gene Ontology) analysis and KEGG (Kyoto Encyclopedia of Gene and Genome) pathway analysis using DAVID (database for annotation, visualization, and integrated discovery) tool. The top 14 KEGG pathways were further assessed, and 19 overlapping genes derived from pathways of hepatitis B and cancer were discovered. The overlapping targets were further mapped in the online tool UALCAN to evaluate the survival rate of hepatocellular carcinoma (HCC) patients. We found that the overexpression of Grb2 (growth factor receptor-binding protein 2) (*p* < 0.0001) was linked to poor overall survival for liver HCC patients, followed by AKT1 (*p* = 0.0015) and PIK3CA (*p* = 0.0088). The present study analyzes the drug-target-disease network and may prove to be a useful tool in gene-phenotype connectivity for genistein in HBV-related liver cancer. Our data also pave the way for further research on Grb2 during the development of chronic HBV infection in liver cancer.

## 1. Introduction

Worldwide, chronic infection due to hepatitis B virus (HBV) is recognized as a major reason for hepatocellular carcinoma (HCC), with a huge financial burden to the society (Sayiner et al., 2019; Villanueva, 2019). In China, primary liver cancer is a commonly occurring malignant tumor and the second leading cause of tumor-related death, among which 85%–90% are HCC, and in nearly 80% cases, HCC is caused by HBV infection (Prevention of Infection Related Cancer [PIRCA] Group, Specialized Committee of Cancer Prevention and Control, Chinese Preventive Medicine Association, 2019). The traditional treatment of HCC is partial hepatectomy and liver transplantation, which has its disadvantages such as low success rate, easy recurrence, and a series of ethical problems caused by the scarcity of liver source (Tang et al., 2018). Therefore, the development of new therapeutic strategies surpassing these limitations is urgently needed. Currently, more and more researchers are focusing on the discovery of more effective anticancer molecules from natural sources.

Genistein is an isoflavone derived from soy and exhibits diverse molecular effects including anti-inflammatory and antioxidative, promotes apoptosis, and modulates metabolic pathways and steroidal hormone receptors (Mukund et al., 2017). This small compound imparts beneficial effects on public health and has garnered great interest as a potential therapeutic agent for oncology research, Alzheimer's and cardiovascular diseases (Hwang et al., 2009; Kaygusuz et al., 2010; Devi et al., 2016). Thus, genistein was investigated quite exhaustively to understand its mechanism of action and identify its primary and secondary targets (Tategu et al., 2008; Katiyar et al., 2015; Devi et al., 2016; Shin et al., 2017). While several research reports on genistein are available, there is a need for a relevant strategy for the systematic study on genistein.

The development of molecular biological techniques, such as high-throughput sequencing and proteomics, has generated a wealth of information on differentially expressed genes or proteins, which presents far more challenges for integration and phenotype. The approach to bypassing this challenge is to apply a network-based technique that has an advantage in analyzing extensive data underlying disease to discover the association between a drug, its targets, and interacting proteins (Barabási et al., 2011; Vidal et al., 2011). Multiple drug-centered databases such as DrugBank have been regarded as a major source for providing comprehensive bioinformatic and pharmacological information from the data extracted from the primary literature. It could also provide an approach to study drugs and their targets at the systematic level because of its quality, breadth, and unique data (Wishart et al., 2017).

In this analysis, we obtained 13 genistein-target proteins from DrugBank, mapped the 13 primary target proteins into the online STRING (Search Tool for the Retrieval of Inter-acting Genes/Proteins) database, and generated 209 secondary protein targets-associated genes and constructed their protein–protein interaction (PPI) network with the Cytoscape software. Primary target genes and the genes associated with them were further analyzed using DAVID (database for annotation, visualization, and integrated discovery) online tools for KEGG (Kyoto Encyclopedia of Genes and Genomes) biochemical pathways, and finally, the top 14 KEGG pathways were picked out. Nineteen overlapping genes derived from hepatitis B and pathways in cancer on the top 14 KEGG pathways were identified. At last, the expression level and overall survival (OS) of these hub genes were analyzed with UALCAN, which is a tool to facilitate the study of survival associations and gene expression variations across tumors based on exhaustive evaluation of TCGA (The Cancer Genome Atlas) gene expression data (Chandrashekar et al., 2017). The approach could help in formulating methodology to treat hepatitis B-related liver cancer with genistein. Therefore, we investigated the relationship between drug targets and therapeutic biomarkers within the background of the human PPI networks.

## 2. Methods

### 2.1. Genistein and its targets search

The online DrugBank is an open source and broad scope database including detailed information on drugs and their targets. Drug-target proteins and related genes were obtained from the DrugBank database, version 5.0.10. It contains 10,555 drugs and more than 200 data field, half of them related to a drug target or protein data (Wishart et al., 2017). The DrugBank database was searched to explore the interaction between genistein and its targets and to generate a genistein-target network in this study. We accessed information about genistein from database using the following fields: “Name,” “Accession Number,” “Groups,” “Categories,” “Indication,” and “Targets.” Detailed data descriptions about “Target protein,” “Gene Name,” “UniProt ID,” “Actions,” “Organism,” and “Gene Name” were extracted in the “Targets” field. Based on the data, we created a visualization chart and made further analysis.

### 2.2. Drug-target PPI network generation/visualization

Based on the use of DrugBank search function, drug primary target proteins were collected for genistein. Then, we mapped the primary target proteins into the online STRING database (Szklarczyk et al., 2015) to generate second-level PPI data. A confidence score above 0.4 and maximum number of interactors no more than 20 were set as the cutoffs. The data were integrated into protein targets-associated genes network and visualized with the Cytoscape software (version 3.6.0).

### 2.3. Functional enrichment analysis

To characterize the biochemical pathways and functions linked to genistein gene, GO (Gene Ontology) enrichment analysis and KEGG pathway enrichment analysis were carried out using an online tool DAVID, which is an online database and analytic tool that provides information about a large list of genes or proteins and their potential functions (Huang et al., 2009). Then, based on GO enrichment analysis and the KEGG biochemical pathways, significant gene sets were arranged and the top 14 pathways were selected with an adjusted *p*-value of <0.01 to explore the obtained information. Finally, we chose 3 of the 14 pathways for further analysis and extracted hub genes in these three pathways.

### 2.4. Survival analysis and level of hub genes expression

A recently developed interactive web resource, UALCAN, is used to analyze cancer transcriptome data from clinical data as well as TCGA level 3 RNA-seq data. It can comparatively analyze the expression of a query gene(s) between normal and tumor samples and assess the effect of clinicopathologic features and level of gene expression on patient survival (Chandrashekar et al., 2017). The expression of hub genes in cancer and normal tissues could be explored with reasonable success. Then, the relationships were visualized through box plot. Furthermore, the *p*-value of survival Kaplan–Meier plotter was estimated and displayed on the plot.

## 3. Results

### 3.1. Characteristic bioactivities of genistein and identification of genistein-target proteins with DrugBank

Genistein was input as a query in DrugBank, and it consisted of 18 categories according to DrugBank category annotation, including anticancer agents, antineoplastic agents, ABCG2/BCRP inhibitors, chromones, benzopyrans, substrates for cytochrome P-450 CYP1A2, estrogens, enzyme inhibitors, non-steroidal, flavonoids, hormones, hormone antagonists and substitutes, inhibitors of P-glycoprotein/ABCB1, isoflavones, phytoestrogens, protective agents, protein kinase inhibitors, and pyrans. The grouping status indicates that genistein is an investigational drug and is currently under clinical trials to assess its effectiveness in treating prostate cancer (Table 1). The 13 proteins that are primary genistein targets include ESRRA, AKT1, ESR2, ESRRB, TOP2A, CYP1B1, PTK2B, NCOA1, ESR1, NCOA2, NR1I2, GPER1, and SHBG (Table 2).

**Table 1.** Characterization of Genistein with DrugBank

Name	Accession number	Groups	Categories	Indication
Genistein	DB01645 (EXPT01582)	Investigational	Anticarcinogenic agents	Currently, genistein is being studied in clinical trials as a treatment for prostate cancer.
			Antineoplastic agents	
			BCRP/ABCG2 inhibitors	
			Benzopyrans	
			Chromones	
			Cytochrome P-450 CYP1A2 substrates	
			Enzyme inhibitors	
			Estrogens	
			Estrogens, nonsteroidal	
			Flavonoids	
			Hormones	
			Hormones, hormone substitutes, and hormone antagonists	
			Isoflavones	
			P-glycoprotein/ABCB1 inhibitors	
			Phytoestrogens	
			Protective agents	
			Protein kinase inhibitors	
			Pyrans	

**Table 2.** Identification of Target Proteins of Genistein with DrugBank

Target protein	Gene name	UniProt ID	Actions	Organism
Estrogen receptor beta	ESR2	Q92731	Not Available	Human
DNA topoisomerase 2-alpha	TOP2A	P11388	Not Available	Human
Protein-tyrosine kinase 2-beta	PTK2B	Q14289	Not Available	Human
Nuclear receptor coactivator 1	NCOA1	Q15788	Not Available	Human
Estrogen receptor alpha	ESR1	P03372	Not Available	Human
Nuclear receptor coactivator 2	NCOA2	Q15596	Not Available	Human
Steroid hormone receptor ERR2	ESRRB	O95718	Agonist	Human
Steroid hormone receptor ERR1	ESRRA	P11474	Agonist	Human
Nuclear receptor subfamily 1 group I member 2	NR1I2	O75469	Not Available	Human
RAC-alpha serine/threonine-protein kinase	AKT1	P31749	Not Available	Human
G-protein coupled estrogen receptor 1	GPER1	Q99527	Not Available	Human
Cytochrome P450 1B1	CYP1B1	Q16678	Not Available	Human
Sex hormone binding globulin	SHBG	P04278	Not Available	Human

### 3.2. Generation and visualization of drug-target networks by STRING database and Cytoscape

**FIG. 1.**
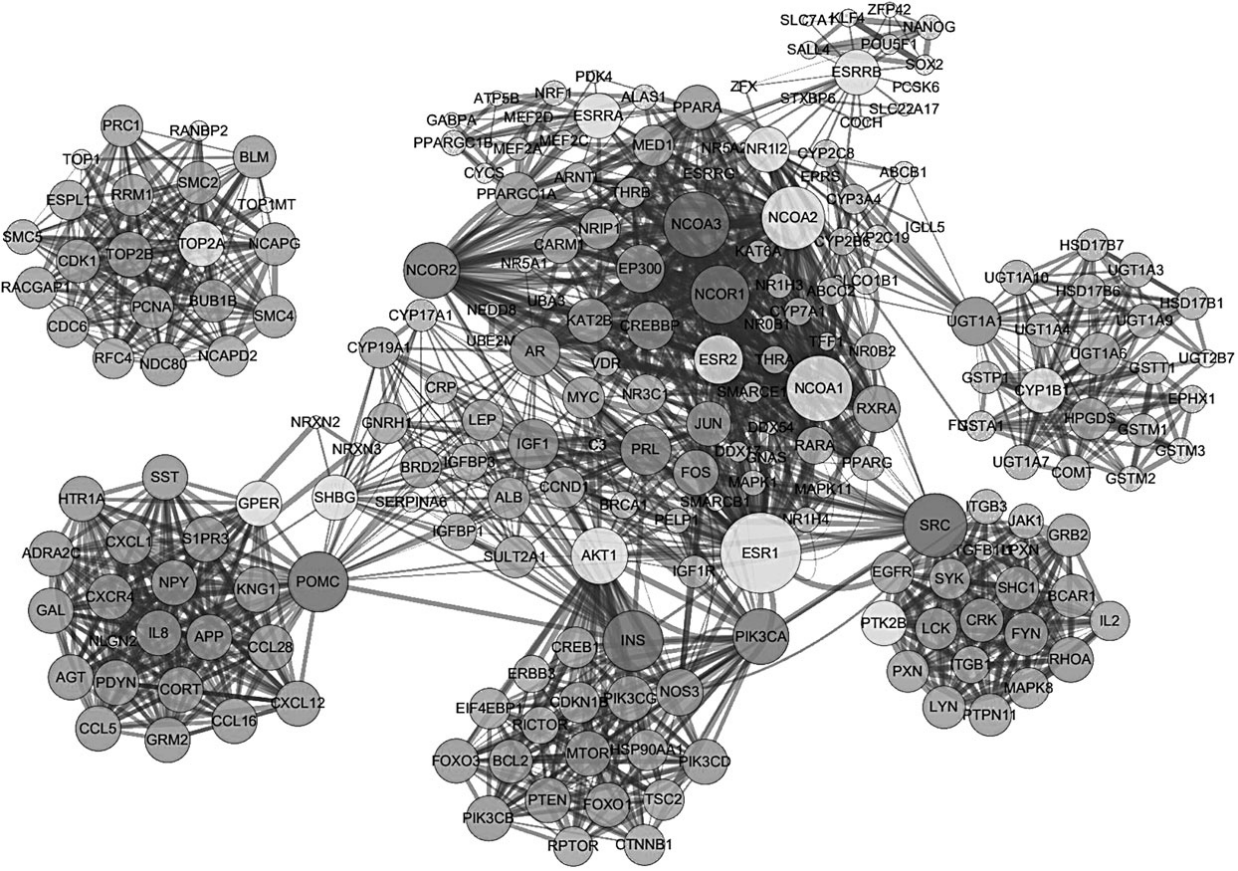
The PPI network of 13 primary protein targets of genistein and 209 secondary protein targets-associated genes. The *light* balls present the primary protein and the *dark* balls stand for the secondary protein targets-associated genes. The balls' diameter suggests the counts, which mean the PPI. PPI, protein–protein interaction.

The STRING protein inquired from public databases identified 209 secondary protein targets-associated genes of 13 primary protein targets for genistein. We made the PPI network of 13 primary protein targets and their secondary protein targets-associated genes with visualization of genistein target-protein interactions by the Cytoscape software (Fig. 1).

### 3.3. Evaluation of functional attributes combined with genistein-mediated variation in gene sets using the GO enrichment analysis and the KEGG biochemical pathways

**FIG. 2.**
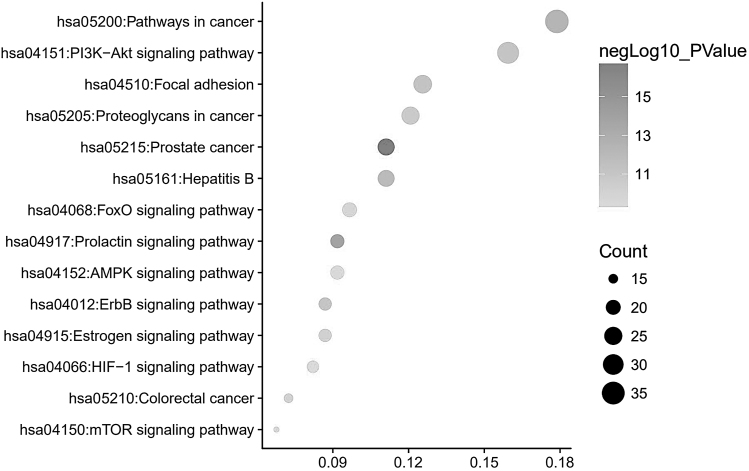
Scatterplot of the KEGG pathway analysis of 13 primary protein targets of genistein and 209 secondary protein targets-associated genes. KEGG, Kyoto Encyclopedia of Gene and Genome.

**FIG. 3.**
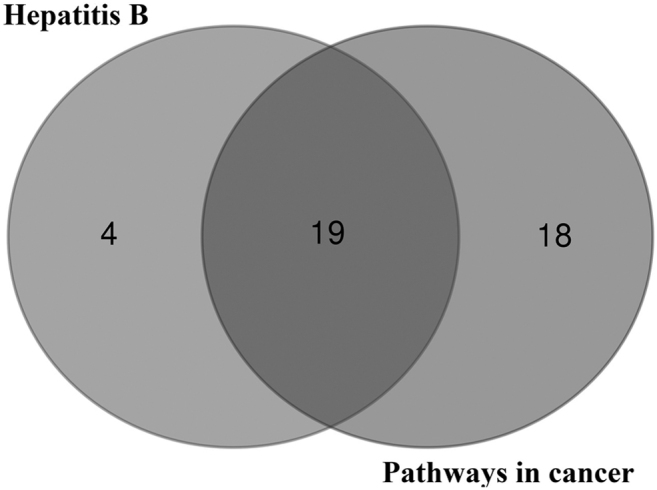
The data of Venn diagram are extracted from hepatitis B and pathways in cancer, and it demonstrate the total number of genes overlapping in the two pathways.

We applied the GO enrichment analysis and the KEGG biochemical pathways with DAVID to assess the functions of genistein-mediated gene sets. On analyzing the top 14 KEGG pathways, genistein-mediated gene sets and their protein-associated genes include prostate cancer (23 genes), prolactin signaling pathway (19 genes), pathways in cancer (37 genes), hepatitis B (23 genes), focal adhesion (26 genes), erbB signaling pathway (18 genes), PI3K/AKT signaling pathway (33 genes), proteoglycans in cancer (25 genes), estrogen signaling pathway (18 genes), colorectal cancer (15 genes), FoxO signaling pathway (20 genes), AMPK signaling pathway (19 genes), mTOR signaling pathway (14 genes), and HIF-1 signaling pathway (17 genes) (Fig. 2). Moreover, the 19 overlapping genes derived from hepatitis B and pathways in cancer were discovered (Fig. 3), such as *PIK3CG*, *CDKN1B*, *PIK3CD*, *FOS*, *MAPK8*, *AKT1*, *MYC*, *MAPK1*, *CREBBP*, *JUN*, *BCL2*, *CCND1*, *PIK3CA*, *PTEN*, *CYCS*, *PIK3CB*, *EP300*, *JAK1*, and *GRB2*.

### 3.4. The expression level and hub genes Kaplan–Meier plotter

**FIG. 4.**
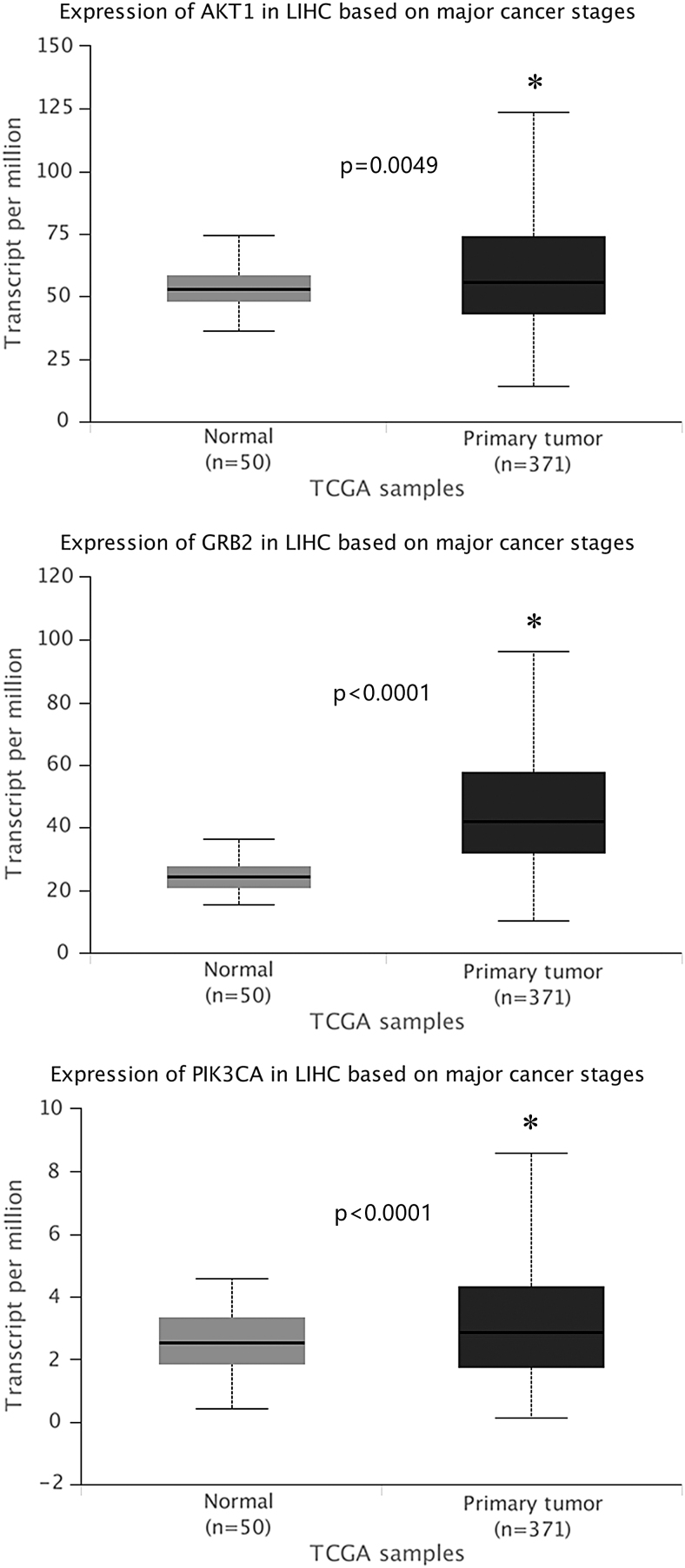
The box plot indicates the expression of the three genes, which are overexpressed in HCC. HCC, hepatocellular carcinoma.

**FIG. 5.**
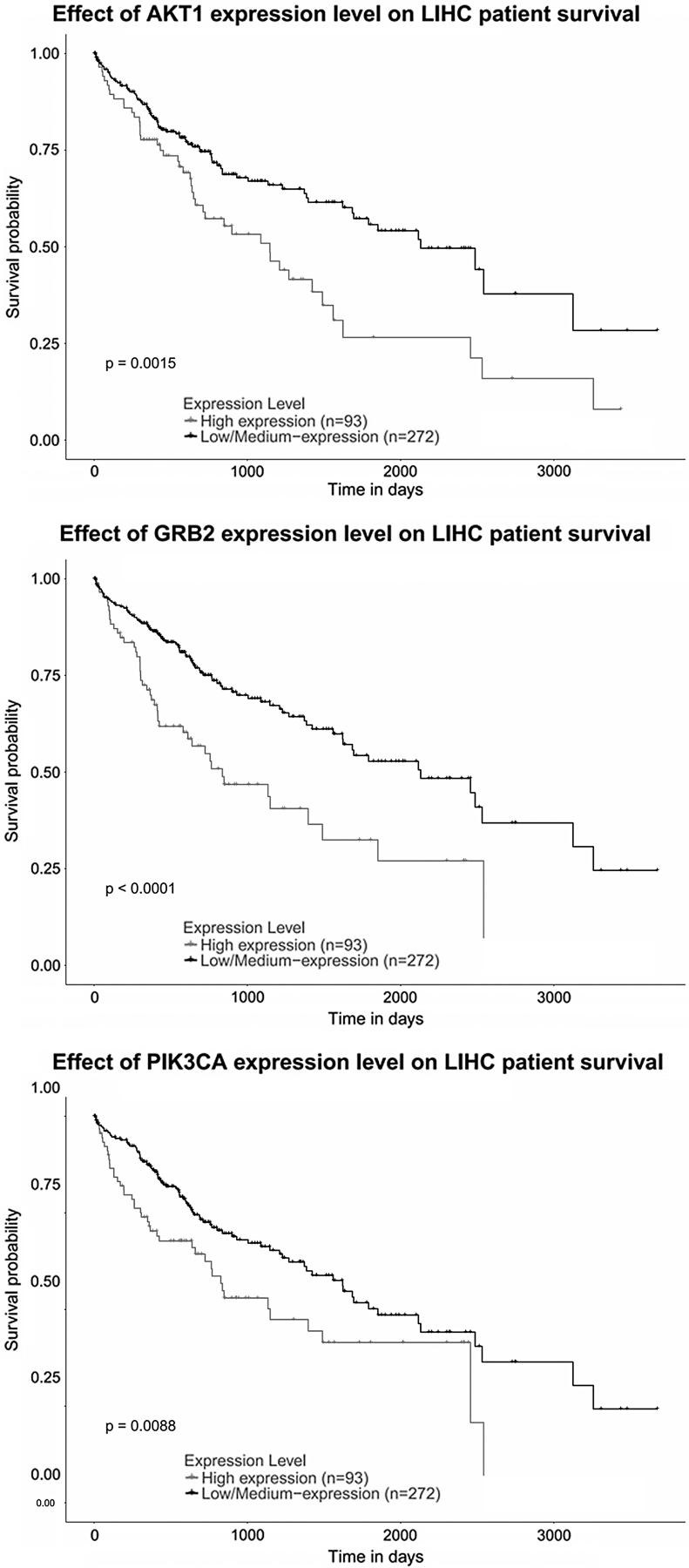
The survival tab demonstrates that overexpression of GRB2, as well as AKT1 (*p* = 0.0015) and PIK3CA (*p* = 0.0088) was linked to worse overall survival for liver HCC patients.

The expression level and prognostic details of 19 selected genes are accessible online (http://ualcan.path.uab.edu). We found that expression of GRB2 (*p* < 0.0001) followed by AKT1 (*p* = 0.0015) and PIK3CA (*p* = 0.0088) was linked to worse OS for liver HCC patients (Fig. 4). The UALCAN web portal also provided information on comparative levels of hub genes expression between cancer and healthy people, and as shown in Figure 5, the level of GRB2 was significantly raised in cancer tissues, based on major cancer stages in cancer patients (statistical significance <1E-12).

## 4. Discussion

Investigations carried out in the past decades have shown that genistein demonstrates synergistic activities combined with chemo-drugs (Spagnuolo et al., 2015). High multifunctional activities and various targets have been confirmed for genistein to explore this bioactive compound with a series of associated diseases and its capacity of activating several signaling pathways in the event and progress of cancer (Mukund et al., 2017). However, the data on the extensive beneficial effects facilitated by genistein remain unsystematic. Thereby, a systematic method for studying genistein and its target proteins is imperative to elucidate the links of their interactome and to observe their biological effects in HBV-related HCC. In this study, we investigated the relationships between genistein and its targets under the background of networks to further illustrate the potential biochemical mechanisms of genistein and its related proteins as well as its clinical effect in HBV-related cancer by online tools.

A system biochemistry approach integrating multiple web-based tools was applied, and associations of HBV-related HCC and biological molecules with drug targets were evaluated. We obtained 13 primary and 209 secondary target genes/proteins in DrugBank and STRING. Nineteen overlapping genes from 2 of 14 enriched KEGG pathways related to genistein-altered genes were selected. The overexpression of AKT1, PIK3CA, and GRB2 in HCC was linked to decline in OS as per UALCAN. Therefore, we hypothesized that those three genes might be potential targets by genistein during the development of chronic HBV infection into liver cancer.

The PI3K/AKT signaling pathway has been found abnormally activated in HCC and affects cell behavior, including proliferation, survival, metabolism, and tumorigenesis (Chen et al., 2001; Cho et al., 2001). Moreover, previous finding indicated that the association of anticarcinogenic properties of genistein is associated with the suppression of PI3K/AKT signaling pathway in HCC (Ma et al., 2011; Wang et al., 2014). Growth factor receptor-binding protein 2 (Grb2) is one of the components of receptor tyrosine kinase signaling pathway, and its expression level can change the activation degree of the pathway, thereby regulating cell growth, differentiation, migration, and so on. Abnormal expression of GRB2 has key part in the occurrence, invasion, transformation, and metastasis of tumors (Frelin et al., 2017). Grb2 has a crucial part in tyrosine kinase-mediated signal transduction that includes linking receptor tyrosine kinases to the Ras/mitogen-activated protein kinase pathway, which is associated with oncogenic outcome. There are some data on the study the mechanisms between genistein and Grb2 protein expression. However, it also remains unclear whether the anticarcinogenic activities of genistein are associated with Grb2 during the development of chronic HBV infection into liver cancer. Hence, the mechanisms of genistein and Grb2 expression in HBV-related liver cancer should be further studied.

In conclusion, with the increase in number of studies on genistein by traditional experimental techniques, more and more genistein targets will undoubtedly be identified. However, there is ambiguity in determining whether the specific biological effects on genistein can be definitively assigned to the individual or simultaneous modulation of their newly established or identified targets. This analysis enabled the interpretation of the inherent mechanisms of HBV-related cancer and genistein's target identification and would ultimately aid in future studies with rational experimental likelihood for detection and validation of logical hypotheses about the role of genistein in HBV-related liver cancers in the future.
